# Dietary differences between elderly Iranians living in Sweden and Iran a cross-sectional comparative study

**DOI:** 10.1186/1471-2458-11-411

**Published:** 2011-05-31

**Authors:** Afsaneh Koochek, Parvin Mirmiran, Kristina Sundquist, Firoozeh Hosseini, Tohid Azizi, Ali S Moeini, Sven-Erik Johansson, Brita Karlström, Fereidoun Azizi, Jan Sundquist

**Affiliations:** 1 Department of Food, Nutrition and Dietetics, Uppsala University, Uppsala, Sweden; 2 Research Institute for Endocrine Sciences, Shahid Beheshti University of Medical Sciences, Tehran, Iran; 3 Center for Primary Health Care Research, Lund University/Region Skåne, Sweden; 4 Stanford Prevention Research Center, Stanford University School of Medicine, California, USA

## Abstract

**Background:**

During the last decades, global migration has increased and many immigrant groups have a higher prevalence than the native born population of several cardiovascular disease risk factors, including poor dietary habits. However, it is uncertain if dietary habits in immigrant populations reflect dietary habits in their country of origin or if the current diet is a consequence of the migration and possible change of dietary habits. The aim of this study was to examine possible dietary differences between elderly Iranians living in Stockholm, Sweden with elderly Iranians living in Tehran, Iran, taking into account sex, age, marital status, and education.

**Methods:**

Dietary intakes were assessed by semi - quantitative food frequency questionnaire in a cross-sectional study of 121 Iranians living in Stockholm and 52 Iranians living in Tehran, aged 60-80. Differences in dietary habits between the two groups was analysed by bootstrapped regression analyses with 1000 replications.

**Results:**

Iranians living in Sweden had significantly higher intake of protein, total fat, fiber than Iranians living in Iran, but lower consumption of carbohydrates. The observed differences in intake of macronutrients were reflected in consumed amount of all food items, which were higher among Iranians living in Iran with the exception of bread and grain consumption which was lower.

**Conclusions:**

There are general differences in dietary habits between Iranians living in Iran and Iranians living in Sweden. Parts of observed differences in dietary habits may reflect a favourable adoption process to the Swedish dietary habits after migration. Meanwhile other differences are point of concern in light of the high prevalence of overweight, among Iranians living in Sweden and can have unfavourable impact in particular in the context of cardiovascular health.

## Background

The world-wide obesity epidemic is alarming because it is strongly associated with dyslipidemia, insulin resistance, the metabolic syndrome,[1] low-grade inflammation [2,3], and diabetes [1], that are important for the development of arteriosclerosis [1-3], and subsequently cardiovascular disease (CVD), one of the main causes of death in both industrialized and developing countries. Although a recent study revealed that mean body mass index (BMI) and the prevalence of obesity appear to have levelled off in the Swedish adult population between 2000/01 and 2004/05 in both men and women [4] another study demonstrated that the magnitude of the obesity problem is particularly high in urban deprived neighbourhoods in for example Stockholm County, Sweden, where Middle Eastern immigrants had a more than 3 times higher odds of being obese than their Swedish-born counterparts [5].

During the last decades, global migration has increased and many immigrant groups have a higher prevalence than the native born population of several CVD risk factors, including poor dietary habits [6-8]. Previous studies have mostly examined dietary habits in middle-aged immigrants and/or compared the dietary habits in immigrant groups with the native born population [9,10]. Although these studies provide valuable information about dietary differences between immigrants and natives, they are unable to detect possible differences in dietary habits between immigrant populations and the population in their country of origin and differences that exist already before the migration. In this study we had the novel opportunity to analyze dietary habits in elderly Iranian immigrants living in Sweden and elderly Iranians living in Iran

Iranian immigrants older than 60 years represent one of the largest group of elderly immigrants in Sweden. We have previously shown that elderly Iranians living in Sweden have a higher mean BMI than Swedes in the same age group [11]. Additionally, as many as 80 percent of elderly Iranian women in both Sweden and Iran have abdominal obesity [12].

It is uncertain if dietary habits in immigrant populations reflect dietary habits in their country of origin or if the current diet is a consequence of the migration and possible change of dietary habits. This study was able to shed new light on this issue. In addition, the focus on elderly Iranians represent a novel contribution, which is of importance especially as the proportion of elderly people including elderly immigrants is increasing in most western countries. Differences in obesity and living conditions between elderly Iranians in Teheran and Stockholm may help us to understand whether the adaptation and acculturation to a new culture is associated with for example a healthy fruit, vegetable and fat intake, knowledge that could be used in health promoting actions. Furthermore, to explore whether groups who migrate have differences in diet that may influence the cardiovascular risk factor profile than among those who stay, in a vulnerable group of people.

The aim of this study is to examine possible dietary differences between elderly Iranians living in Stockholm, Sweden with elderly Iranians living in Tehran, Iran, taking into account sex, age, marital status, and education.

## Methods

### The study Population

#### Iranians living in Sweden

All Iranian-born persons aged 60-84 settled in the township of Kista, Stockholm (N = 270), were invited to participate in the study via a letter written in both Swedish and Farsi. One hundred and seventy-six persons (65 percent) agreed to participate in the study. Interviews were conducted face to face in Farsi, using a questionnaire based on material produced for the Swedish Annual Level of Living Survey by Statistics Sweden [13].

The non-response analysis, which was followed up by phone call to persons who refused to participate, showed no significant differences between participants and non-participants with regard to sex, education and self-reported weight and height. However, the non-participants were slightly older (p < 0.05) (mean age = 72.8 ± 7.7 years) than the participants (mean age = 70.5 ± 7.0 years).

In order to have the same age group as the Iranian group living in Iran, we excluded four men and 12 women older than 80 years. Of the 160 who participated, 8 had no dietary data. Thus, 152 individuals remained in the final sample.

#### Iranians living in Iran

The reference group consisted of 52 Iranians aged 60-80, within the framework of Tehran Lipid and Glucose Study (TLGS), a prospective study performed on residents of district 13 of Tehran with the aim of determining the prevalence of non-communicable disease risk factors and developing a healthy lifestyle to improve these risk factors [14]. However the Iranian population in Iran is rather young; in 2005 in Iran, the elderly population aged 60 and older was 6.5 percent of the whole population [15]. In addition in the TLGS population which included representative sample of 15005 participants aged 3 years and over of the urban population of Tehran, 12 percent were 60 years and older. Furthermore 1476 persons were selected randomly for dietary assessment in the TLGS population. 52 subjects (3.5 percent) aged 60 years and over were willing and able to participate in the dietary survey and all are included in this study.

#### Dietary intake

A semi-quantitative food frequency questionnaire (FFQ) for the elderly Iranians living in Stockholm was adapted from the dietary survey of the Swedish population which was carried out by the Swedish National Food Administration in collaboration with Statistics Sweden [16]. The FFQ was originally designed to assess dietary intake in terms of energy and macronutrient intake (total fat, carbohydrate and protein) as well as intake of different food groups, and was modified to exclude prepared dishes and typically Swedish food that are not commonly eaten by Iranians. The modified FFQ consisted of a list of 114 food items and the standard serving size for each item. The FFQ was conducted in a day centre for Iranian seniors in Kista or in the participant's home face-to-face during 30 minutes by the first author (AK) of the present study. AK is familiar with the Persian cuisine and language. If a male participant were unable to describe the content of the food, we ascertained the content by asking the wife or a daughter. Photos from a portion guide [17] were shown to the participants in order to estimate portion sizes of the following food items: bread, cooked rice, mixed salad, fat spread on bread, feta cheese, and cooked pasta. Frequency of consumption was obtained by asking open-ended questions on how often a particular food item was consumed, i.e. per day/week/month or during the last year. Participants were also asked about the fat percentage of dairy products and margarine. Brand name of items like bread, jam, breakfast cereal, cookies/cakes, fruit juices and ice cream were asked to obtain a close match with items listed in the Swedish food tables. An open question at the end of the interview allowed individuals to report any other eaten foods not listed in the FFQ. The 114 food items were divided into 18 food categories, which were combined into six major food groups that matched the TLGSs classification of food groups (Table 1) [18]. Items that did not fit into any of the six groups were individually categorized (ice-cream, jam, nuts, sugar, sweets, potato chips, popcorn, cheese doodles, chocolate, honey, soft beverages and alcoholic beverages) and assessed for their content of macronutrients. Based on the reported amounts we calculated daily intakes of macronutrients and each food item in grams. Daily grams were computed for each food item as the daily frequency, e.g. monthly and weekly frequencies were divided by 30 and 7, respectively.

**Table 1:** Food groupings in dietary pattern analysis

Food groups	Food items
Bread and grain	Refined and whole bread and grain, pasta, cookies/cakes, breakfast cereal, porridge, rice
Vegetables and roots	Vegetables, legumes, potato, starchy vegetables
Fruit	Fruit, fruit juice, berries, melons
Meat	Red meat, poultry, fish, egg, offal
Dairy	Milk, fermented milk, yoghurt, cheese
Edible fat	Vegetable oil and animal fat

Data obtained from the FFQ were entered into DIETIST XP software [19], which is based on food-tables from the Swedish National Food Administration [20] that includes almost 1600 items and 50 nutrients.

Doubly-labeled water method can accurately evaluate total energy expenditure and can be use for assessing the validity of reported total energy intake [21,22]. However, because of the high cost of this method, validation of reported dietary intakes are most frequently measured by Goldberg cut-off method which can be used to evaluate the mean population bias in reported energy intake [23]. Therefore, for each participant in Sweden, the FFQ was validated by the Goldberg cut-off [24], which was revised in the year 2000 [23]. The model is based on reported energy intake (EIrep), estimated basal metabolic rates (BMRest), and physical activity level (PAL).

BMRest was estimated from the Schofield equations [25] for persons older than 60 years as follows:

Data on self-reported leisure-time physical activity was recorded during the interviews, and subjects were categorized into 5 levels of physical activity: very light (almost no activity at all), light (walking, non-strenuous cycling or gardening approximately once a week), moderate (regular activity at least once a week, e.g., walking, bicycling, or gardening; or walking to work 10-30 min/d), active (regular activities more than once a week, e.g., intensive walking, bicycling, or sports), and very active (strenuous activities several times a week). PAL was systematically estimated for each subject according to a method developed by Johansson et al. [26].

Subjects were identified as under-reporters, acceptable-reporters, or over-reporters according to their EIrep/BMRest and the lower and upper cut-offs which were calculated as follows [23]:

Where s.d. min is - 2 for the 95 percent lower confidence limit, s.d. max is + 2 for the 95 percent upper confidence limit. S is the factor that accounts for the variation in EI, BMR, and PAL and is given by:

CVwEI is the within-subject coefficient of variation (CV) in EI, d is the number of days of diet assessment, CVwB is the CV of repeated BMR measurements, and CVtP is the total CV in PAL. CVtP is the CV derived from the mean and standard deviation of a study and includes true between-subject variation, an element of within-subject variation and methodological errors. The CVs used were 23 percent for CVwEI, 8.5 percent for CVwB and 15 percent for CVtP [16]. Subjects were defined as under-reporters if the ratio EIrep/BMRest was below the lower 95 percent confidence limits (< 0.56), acceptable-reporters if the ratio was within the lower 95 percent confidence limits and the upper 95 percent confidence limits (0.56 - 1.78), and over-reporters if the ratio was above the upper 95 percent confidence limits (> 1.78). Approximately 80 percent of the participants were acceptable-reporters, 14 percent were over-reporters, and 6 percent were under-reporters. After exclusion of subjects who were not acceptable reporters, 121 subjects (41 men and 80 women) remained in the final data analysis.

##### Validation of FFQ in Iran

Usual dietary intake among elderly Iranians living in Tehran was assessed with the use of a 168-item FFQ and was administered by trained dietitians. The FFQ consisted of a list of foods with a standard serving size. Participants were asked to report their frequency of consumption of each food item during the previous year on a daily (eg, bread), weekly (eg, rice, meat), or monthly (eg, fish) basis. Each food and beverage were then coded according to the prescribed protocol and analyzed for content of energy and the other nutrients by using Nutritionist III software (version 7.0; N-Squared Computing, Salem, OR), modified for Iranian foods. The validations process of the FFQ which was used in Tehran indicates that the FFQ provides reasonably reliable measures of the average long-term dietary intakes [27,28].

### Definition of sociodemographic and anthropometric variables

*Migration status *was defined as Iranians living in Iran or in Sweden.

*Age *was categorized into the following groups: 60-64, 65-69, 70-74 and 75-80 years.

*Educational status *was used as an indicator of socioeconomic status; the participants were dichotomized into: (1) ≤ 9 years of education and (2) > 9 years of education.

*Marital status *comprised two groups: married or widowed.

*Anthropometric measures *included weight (kg), height (m) and waist circumference (cm). General obesity was measured with body mass index (BMI, kg/m²), calculated from weight/height². Overweight and general obesity was defined as 25 ≤ BMI < 30 and BMI ≥ 30, respectively.

### Statistical analysis

Dietary intakes were adjusted for total energy by calculating residuals from regression analyses, with total energy intake as the independent variable, and each of the macronutrients, fiber, and food groups as the depended variable [29]. Stata version 10 was used for the statistical analysis [30]. All outcome variables (Energy, macronutrients and each food item intake) were subjected for transformation because of non-normally distribution. However, neither logarithm nor Box-Cox transformation [31] improved the non-normally distribution. Therefore, we used Bootstrap regression analyses with 1000 replications to examine possible Dietary differences between elderly Iranians living in Stockholm, and elderly Iranians living in Tehran [32]. By bootstrapping correct confidence intervals for the beta-coefficients will be obtained. We show a linear main effect model adjusted sex, age, education, and marital status. We also show means (and 95% confidence intervals) adjusted for the same variables as in then regression analyses.

Intake of macronutrients, fiber and food items were analyzed as continuous variables. The following reference categories were chosen: Iranians living in Iran (migration status), 60-64 years (age), women (sex), ≤ 9 years (education), and married (marital status). The results are shown as β-coefficients with 95percent confidence intervals (CIs). Two models were taken into consideration: the first model included sex and age in the analysis, whereas the main model included all the independent variables simultaneously i.e. sex, age, education and marital status. The interactions between the independent variables were also tested.

### Ethical Considerations

This study was approved by the Karolinska Institute Ethics Committee (Register number: 92/03, March 10, 2003). The Ethical Committee of the Endocrine Research Center approved the Tehran Lipid and Glucose Study. All participants gave their informed consent to participate in the two studies.

## Results

### Characteristics of the sample

Sociodemographics and anthropometric characteristics of the study participants by migration status are shown in Table 2. The mean age of Iranians living in Iran was lower than Iranians living in Sweden. Iranians living in Sweden were more often widowed than Iranians living in Iran. The mean BMI was similar in Iranians living in Iran and Iranians living in Sweden, and is indicative of overweight status (Table 2).

**Table 2:** Characteristics of the study population, by migration status of Iranians living in Iran† and Iranians living in Sweden‡, aged 60-80 years

	Migration status	
Characteristics	Iranians in Iran (n = 52)	Iranians in Sweden (n = 121)
Sex (%)		
Women	40.4	66.1
Men	59.6	33.9
Age Group, years (%)		
60-64	57.7	28.1
65-69	32.7	28.1
70-74	7.7	26.5
75-80	1.9	17.4
Mean age (SD) years	64.4 (4.0)	68.6 (5.9)*
Education (%)		
> 9 years	50.0	47.1
≤ 9 years	50.0	52.9
Marital status (%)		
Married	86.5	48.8
Widowed	13.5	51.2
BMI (kg/m²) Mean (SD)	27.8 (4.3)	27.9 (4.7)
Overweight (%)		
(25 ≤ BMI < 30)	38.5	47.1
Obesity (%)		
(BMI≥30)	25.0	28.9

SD - Standard deviation; BMI - body mass index, * significantly different *p*< 0.05, †TLGS. Tehran Lipid and Glucose study 2005. ‡Study of elderly Iranians living in Stockholm 2004-2

### Dietary intake

Iranians living in Sweden had a higher consumption of protein, fat and fiber, than Iranians living in Iran, but lower consumption of carbohydrates. The observed differences in intake of macronutrients were reflected in consumed amount of all food items, which were higher among Iranians living in Iran with the exception of bread and grain consumption which was lower (Table 3).

**Table 3:** Average energy adjusted daily intake of macronutrients, fibre, and selected food items among Iranians living in Iran and Sweden by migration status and sociodemographic and anthropometric characteristics

Migration status, sociodemographic and anthropometrics characteristics	Category	Protein g/day	Carbohydrates g/day	Fat g/day	Fibr g/day	Bread and grain g/day	Vegetables and roots g/day	Fruit g/day	Meat g/day	Dairy g/day	Edible fat g/day
**Iranians in Iran (n = 52)**	**All**	67	347	73	10	352	315	221	89	273	29
Sex	Women	66	347	72	12	370	282	267	76	298	32
	Men	68	348	74	8	339	338	189	98	256	26
Education	> 9 years	66	347	73	7	345	292	249	98	307	31
	≤ 9 years	69	348	74	12	359	339	194	81	239	26
Marital status	Married	67	342	76	8	335	322	228	92	295	29
	widowed	68	380	55	19	455	270	179	70	139	29
Anthropometry	25 ≤ BMI < 30	67	347	74	8	347	307	193	86	275	29
	BMI≥30	68	348	73	15	335	286	293	78	351	28
**Iranians in Sweden (n = 121)**	**All**	83	267	97	26	272	429	442	168	454	52
Sex	Women	84	270	97	28	272	495	449	169	471	54
	Men	81	260	97	22	271	300	430	165	421	49
Education	> 9 years	84	270	94	26	279	437	431	168	449	48
	≤ 9 years	82	265	100	26	265	421	452	168	459	55
Marital status	Married	82	265	97	25	270	448	419	166	414	50
	widowed	84	269	98	27	274	411	464	170	493	54
Anthropometry	25 ≤ BMI < 30	82	265	97	26	275	436	425	162	449	49
	BMI≥30	83	265	101	25	249	401	443	166	505	55

Table 4 shows means and bootstrapped β-coefficient with 95% confidence intervals for energy adjusted intake, of macronutrients fiber and selected food items among Iranians living in Iran and Sweden adjusted for sex, age, education and marital status. The reference is given the value of zero so that the value of the β-coefficient corresponds to the difference in gram for all macronutrients, fiber and food items compared to the reference category. The results shows that Iranians living in Sweden had significantly higher intake of protein, total fat, fiber and all food items with the exception of carbohydrates and bread and grain intake which were lower among Iranians living in Sweden.

**Table 4:** Means and bootstrapped β-coefficient* with 95% confidence intervals (CI) for energy adjusted intake, of macronutrients, fibre, and selected food items among Iranians lining in Iran and Sweden adjusted for sex, age, education, and marital status

	Adjusted means		Main model	
Dietary variables	Mean	CI	β-coefficient	CI
Protein				
Iranians in Iran	65.7	61.9-69.6	0	Reference
Iranians in Sweden	83.6	80.8-86.3	17.9	13.1 - 22.7
Carbohydrates				
Iranians in Iran	351.7	338.7-364.7	0	Reference
Iranians in Sweden	265.3	256.0-274.5	-86.4	-104.5 - -68.4
Fat				
Iranians in Iran	74.7	66.0-79.4	0	Reference
Iranians in Sweden	97.4	93.0-101.7	24.6	15.9 - 33.4
Fiber				
Iranians in Iran	10.2	6.2-14.1	0	Reference
Iranians in Sweden	25.7	23.7-27.7	15.5	10.8 - 20.2
Bread and grain				
Iranians in Iran	350.0	318.1-381.9	0	Reference
Iranians in Sweden	272.5	252.8-292.3	-77.4	-117.5 - -37.4
Vegetables and roots				
Iranians in Iran	310.0	235.1-384.8	0	Reference
Iranians in Sweden	430.1	368.8-496.5	120.2	24.6 - 215.7
Fruit				
Iranians in Iran	234.2	180.3-288.1	0	Reference
Iranians in Sweden	435.4	391.1-479.7	201.2	130.7 - 271.6
Meat				
Iranians in Iran	83.7	70.9-96.5	0	Reference
Iranians in Sweden	170.1	159.4-180.9	86.4	69.0 - 103.8
Dairy				
Iranians in Iran	285.6	202.7-368.4	0	Reference
Iranians in Sweden	448.4	408.8-487.9	162.8	70.5 - 255.1
Edible fat				
Iranians in Iran	31.7	27.1-36.4	0	Reference
Iranians in Sweden	50.6	46.5-54.6	18.9	12.5 - 25.3

* The reference is given the value of zero so that the value of the β-coefficient corresponds to the difference in gram for all macronutrients, fibre, and food items compared to the reference category.

Analysis of a total 20 first order interaction was simultaneously tested in each of the 10 models. But only an interaction between sex and migrations status (using Iranian women living in Sweden as reference group) for consumption of vegetables and roots was significant and showed that Iranian women living in Sweden had the highest consumption of vegetables and roots (data not shown).

## Discussion

The main finding of this study indicates that there are general novel differences never reported before in dietary habits between Iranians living in Iran and Iranians living in Sweden. These include a reduced intake of carbohydrates, in particular bread and grain products, and an increase consumption of total fat, protein, fiber, fruit, vegetable, meat, dairy and edible fat, among Iranians living in Sweden. These differences remained after accounting for possible confounders.

Our findings agreed with previous migrant studies which indicate that migration especially the adaptation and acculturation to a new culture have an important impact on behavioural factors such as dietary habits [6,7,33]. However the focuses of these studies have been in middle-aged immigrants, which make it difficult to know if the same dietary patterns also are relevant in elderly people who are more likely to adhere to traditional dietary patterns [34].

### Possible pathways

#### Migration and dietary habits

Role of migration process on health behaviour is likely to be associated with adoption processes to positive and negative lifestyles of the host country [10,33,35]. In Sweden, the elderly population, like others in the general Swedish population has nutritionally adequate diets in relation to recommended levels [36]. Hence higher intake of fruit, vegetables, fiber and dairy products among Iranians living in Sweden, can be a part of the adoption process to the Swedish dietary habits. This level of fruit and vegetable consumption equates with current Swedish food administration who recommends to eat 500 games of fruit and vegetables every day [37]. Moreover, the observed lower intake of carbohydrates among Iranians living in Sweden was reflected in lower amount of bread and grain consumed by this group. However, the fact that Iranians living in Sweden had higher fiber intake than Iranians living in Iran, suggest that the major part of the consumed fiber in Sweden should be a result of high consumption of fruit and vegetable which are major sources of soluble fiber. Moreover consumption of soluble fiber is associated with positive effect on plasma LDL-cholesterol, glucose level and decreased risk for cardiovascular disease [38].

Higher total fat and lower carbohydrate consumption suggests that Iranians living in Sweden have adopted dietary habits that are not in line with current nutritional recommendation in Sweden. A similar pattern of edible fat consumption was also observed in a study from Norway indicating that Pakistani women in Oslo substitute butter/ghee with margarine or vegetable oil because they believed that vegetable oil has less fat than butter [39]. For this reason we argue that the observed edible fat consumption can be due to misunderstanding about the beneficial effect of vegetable oil (e.g. olive and canola oil) among Iranians living in Sweden who may believe that vegetable oil is low fat and does not increase weight. Higher total fat intake including fat rich foods such as meat products and dairy is a point of concern in light of the high prevalence of obesity, among Iranians living in Sweden and can have an unfavourable impact on cardiovascular health of this immigrant group.

Food choice might also be influenced by limited economic resources among low-income groups [40,41], which in turn leads to fat rich and energy dense dietary options, which are inexpensive and good tasting [42]. This may explain the high consumption of fat rich food items such as meat and dairy products among Iranians living in Sweden with very low incomes. Because most of Iranians living in Sweden in this study arrived in Sweden late in life and can therefore not be eligible for full retirement pension. However, the explanation that costs is more relevant than nutritional concerns on personal dietary options, contrast with the Norwegian study which indicate that food prices were not the most important predictor of dietary changes among immigrant women [39]. Though it is difficult to determine factors that may influence food choice among this group of immigrant in Sweden with different dietary habits compared to the population of their origin country.

### Limitations and strengths

This study has several limitations that should be considered when interpreting our findings. First, comparing data from two different study samples in different countries with differences in sampling and administration procedures can be associated with variations in validity of data and methodological problems. Therefore, the observed dietary differences between the Swedish and Iranian groups could be due to small differences in used FFQs rather than being real differences. It is for example possible that the much higher fruit intake in Sweden is due to more detailed questions on fruit in the Swedish FFQ than in the Iranian FFQ, on the other hand if the questions on fruit are almost identical in the two FFQs it is likely that the groups differ in fruit intake. However, a similar pattern of adequate consumption of fruit and vegetable was also observed among elderly immigrants in Canada [43], USA [44] and younger non-Western immigrants in Denmark [45].

Second, because of the cross-sectional design of this study, no cause-effect conclusions can be drawn. Third, we had no possibility to include physical activity variable in analysis, because of lack of data on physical activity among Iranians living in Iran. Since physical activity is one of the most important components of between-person variation in energy intake [29], it would be important to include physical activity in future analysis. Finally, although the Stockholm sample is small, it is representative for elderly Iranians living in an urban area, because it is based on the total population of elderly Iranian -born people, living in Kista, where the largest proportion of Iranian-born persons aged 60-84 years in Stockholm County live. In addition we compared elderly Iranian in the largest city in Sweden and elderly Iranians in the largest city in Iran. Moreover, the Tehran Lipid and Glucose Study (TLGS) consist of a population which represent Tehranian population [14]. Only 12% of TLGS and other Tehran population are in age groups 60-84 years, which explains the low number of participants in this study.

Despite these limitations, the present study has several strengths. In contrast to normal parametric methods, the bootstrap method used had the advantage of analyzing the dataset presented here, regardless of skewed distributions of all outcome variables and low sample size. Moreover, the bootstrap method provided analysis without transforming the data so that results can be interpreted directly based on the original measurements, compared with interpreting results in terms of the geometric mean based on transformed nutrient data. Furthermore, our findings may help dietitians, and particularly district nurses and primary health care physicians who during one-year see 70% of the population, to identify factors that influence alteration in dietary habits after migration. Understanding the associations between migration and health behaviors, such as diet, is one important step toward improving overall health status. Our finding that migration especially the adaptation and acculturation to a new culture was positively associated with fruit, vegetable and fat intake suggests that special efforts to promote maintaining fruit and vegetables consumptions and efforts to limit fat consumption should be included into nutrition counseling among this group of elderly immigrants.

These results may also help to plan education programs which aim to increase nutritional knowledge among immigrant groups, which in turn leads to adequate food choices and dietary habits.

## Conclusions

There are general differences in dietary habits between Iranians living in Iran and Iranians living in Sweden. Parts of observed differences in dietary habits may reflect a favourable adoption process to the Swedish dietary habits after migration. Meanwhile other differences are point of concern in light of the high prevalence of overweight, among Iranians living in Sweden and can have unfavourable impact in particular in the context of cardiovascular health.

## Abbreviations

CVD: Cardiovascular disease; BMI: body Mass Index; CI: Confidence Interval; TLGS: Tehran Lipid and Glucose Study

## Competing interests

The authors declare that they have no competing interests.

## Authors' contributions

AK is the corresponding author of the manuscript. She contributed as a principal researcher and writer, including drafting of the article and the analysis and interpretation of the data.

PM, FH, TA, and ASM participated in the conception and design of the TLGS study in Iran and revised the manuscript.

KS participated in drafting and revised of the manuscript for important intellectual content.

SEJ contributed to the study design, performed the statistical analysis, supervised the study, and made substantial contributions to the interpretation of the data.

FA contributed material and participated in design and coordination of the TLGS study in Iran. He revised the manuscript for important intellectual content and final approval.

BK participated in its design of the study, supervised the study and revised the manuscript for important intellectual content.

JS made contributions to the design, acquisition, and interpretation of the data and participated in the writing process by commenting on the manuscript. He supervised the study and gave final approval of the version to be published

All authors read and approved the final manuscript.

## Pre-publication history

The pre-publication history for this paper can be accessed here:

http://www.biomedcentral.com/1471-2458/11/411/prepub
